# Rosettes in T-cell pseudolymphoma: a new dermoscopic finding^☆^^☆☆^

**DOI:** 10.1016/j.abd.2020.05.010

**Published:** 2020-11-16

**Authors:** Rodrigo Gomes Alves, Patricia Mayumi Ogawa, Mílvia Maria Simões e Silva Enokihara, Sergio Henrique Hirata

**Affiliations:** Department of Dermatology, Universidade Federal de São Paulo, São Paulo, SP, Brazil

**Keywords:** Dermatology, Dermoscopy, Pseudolymphoma

## Abstract

Rosettes are small white structures visible with polarized light dermoscopy, whose exact morphological correlation is not yet defined. These small shiny structures are found in several conditions such as scarring, dermatofibroma, molluscum contagiosum, squamous cell carcinoma, basal cell carcinoma, melanoma, melanocytic nevus, discoid lupus erythematosus, and papulopustular rosacea. In this novel report, the authors describe the presence of rosettes in a T-cell pseudolymphoma lesion.

## Introduction

Rosettes are small white structures grouped in the shape of a four-leaf clover, visible only under polarized light dermoscopy. While the literature presents some hypotheses, their exact morphological correlation is not yet defined. Initially, the presence of rosettes corroborated the diagnosis of actinic keratosis and squamous cell carcinoma; however, these structures were later described in several other conditions.^1^, ^2^ In this report, the authors expanded the number of diseases associated with rosettes after finding them in T-cell pseudolymphoma.

## Case report

**Figure 1:**
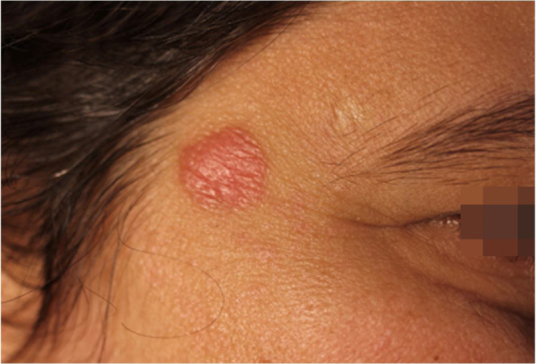
Circular, erythematous plaque, with sharp edges and smooth surface, approximately 1.2 cm in diameter, on the right temporal region.

**Figure 2:**
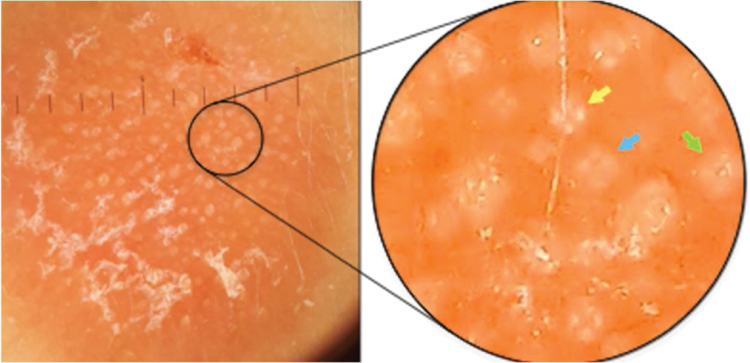
Contact dermoscopy with polarized light, without immersion, with groups of rosettes of about 0.2 mm, oriented in the same direction, interspersed with white dots. In the highlight, there is a well-formed rosette (yellow arrow), a rosette outline (blue arrow), and a white dot (green arrow).

42-year-old female, under follow-up with a genetics and neurology team for suspected Angelman syndrome. She had been using lamotrigine 50 mg/day for two years and clonazepam 1 mg/day. She had a history of a lesion on the right temporal region for six months, with progressive, painless growth, with no pruritus and no report of an insect bite at the site (Fig. 1). On clinical examination, she presented a circular plaque, with sharp edges, measuring 1.2 cm in diameter, with a smooth surface, on the right temporal region. The dermoscopic examination with contact polarized light, without immersion, showed a salmon-colored background, discrete flaking, with white dots interspersed with groups of rosettes of about 0.2 mm, oriented in the same direction (Fig. 2). The biopsy of the lesion center was then performed with a 4-mm punch.

**Figure 3:**
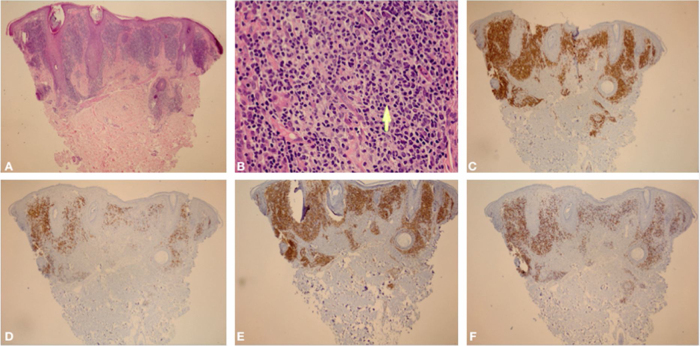
(A), Skin biopsy showing an intense lymphocytic infiltrate filling the interstitial region of the papillary dermis and part of the superficial reticular dermis, predominantly in the upper part of the dermis (Hematoxylin & eosin, ×40). (B), In the highlight, there is a predominance of small lymphocytes with slight nuclear atypias (slight increase in nuclear volume and slight irregularity in nuclear contours) in addition to areas containing plasma cells and histiocytes (Hematoxylin & eosin, ×400). (C), (CD3 – ×40) Immunohistochemical exam showing a predominance of T lymphocytes CD3 positive. (D), (CD20 – ×40) Immunohistochemical exam showing CD20 immunoexpression (B lymphocytes) in part of the infiltrate. (E), (CD4 – ×40) A slight predominance of CD4 positive T lymphocytes can be observed in the infiltrate. (F), (CD8 – ×40) CD8 showing less immunoexpression than CD4.

Histological examination showed an atypical lymphoid proliferation, with intense lymphocytic infiltrate filling the papillary and superficial reticular dermis, notably in its upper portion, with a predominance of small lymphocytes with irregular nuclei and increased volume (Fig. 3A-B). The immunohistochemical examination revealed a predominance of T lymphocytes reactive with CD3, CD4, and a lower expression of CD8; B lymphocytes were detected in part of the infiltrate (Fig. 3C-F) and there was no immunoexpression of CD30. This pattern favors the diagnosis of T-cell pseudolymphoma.

## Discussion

Rosettes can be found in dermoscopy with polarized light in melanocytic and non-melanocytic lesions, but they are not pathognomonic of any dermatosis.^3^, ^4^ They were described in 2009, initially associated with actinic keratosis, lichen planus-like keratosis, and squamous cell carcinoma.^1^ In a series of 6,108 *ex vivo* dermoscopies, the presence of rosettes was verified in several conditions such as scars (6.4%), dermatofibroma (6%), molluscum contagiosum (5.9%), squamous cell carcinoma (4.0%), basal cell carcinoma (1.7%), melanoma (1.4%) and nevi (0.7%).^2^ There are also isolated reports of the presence of rosettes in a lesion of discoid lupus erythematosus and in papulopustular rosacea.^5^, ^6^ To the best of the authors’ knowledge, this is the first report of the presence of rosettes in T-cell pseudolymphoma, but this finding may not be reproducible in other similar cases.

Unlike other crystalline structures, the rosettes do not present angle-dependent enhancement; therefore, when rotating the dermoscope on its central axis, the rosette remains static and does not follow the rotation of the instrument. The rosettes are not all the same size; the small ones (0.1–0.2 mm) appear to correspond to the dispersion of polarized light when in contact with follicular openings filled with opaque material or even with eccrine ducts at the level of the infundibulum.^7^ In turn, larger rosettes (0.2–0.5 mm) would be the result of concentric fibrosis around the follicles.^2^ This diversity in size can be found in the same lesion, and several white dots in between are common.^5^

The white dots were described by Kossard in 1993 in contact dermoscopy with immersion without polarized light on the scalp of a patient with the hypothesis of pseudopelade or lichen planopilaris.^8^ In that study, it was postulated that white dots were the result of a focal decrease in the epidermal melanin underlying fibrous tracts. These findings were later related to several other conditions, including cutaneous lymphocytoma.^9^, ^10^ The authors believe that rosettes and white dots are part of the spectrum of the same process, as suggested in Fig. 2, in which there is a mixture of well-formed rosettes that appear to gradually change into white dots (yellow, blue, and green arrows, respectively). The authors also postulate that many of the white dots described without polarized light are actually rosettes, which would be seen in a thorough analysis with polarized light dermoscopy. However, this clinical observation still lacks histological confirmation.

The dermoscopic findings of primary cutaneous B-cell lymphomas are not specific. In an observational study by Geller et al., 58 dermoscopic images of primary cutaneous B-cell lymphomas were evaluated; salmon backgrounds were observed in 79.3% of the images and prominent vessels in 77.2%. Mascolo et al. observed white circular structures in 90% of cases (total n = 10).

Primary cutaneous T-cell lymphomas represent more than 75% of cutaneous lymphomas. Dermoscopic findings are better defined in cases of mycosis fungoides (MF). In studies by Bosseila et al., small linear vessels were found in 93% of cases of MF, in addition to sharp vessels in 55% and yellowish-orange areas. Lallas et al. also observed these findings, in addition to vascular structures shaped as spermatozoa, with high specificity for MF when compared with dermoscopy of chronic dermatitis lesions.

The studies on dermoscopy of pseudolymphomas are limited to case reports, such as the description of linear vessels seen as whitish reticular lines in cutaneous lymphocytoma by Namiki et al. Arborizing vessels and multiple yellow follicular and perifollicular dots were described by Fujimura et al. in 2012 in cases of pseudolymphomatous folliculitis. In the case of acral pseudolymphomatous angiokeratoma, irregular linear vessels have been described, in addition to white and pink areas with a rainbow pattern at the periphery (Pinus et al.). In observational studies, such as that by Navarrete-Dechent et al., the dermoscopic findings of pseudolymphomas were analyzed together with those of primary cutaneous lymphomas, suggesting that both share dermoscopic similarities, such as an orange background (71.4%), follicular plugs (85%), and linear vessels (78.5%).

**Figure 4:**
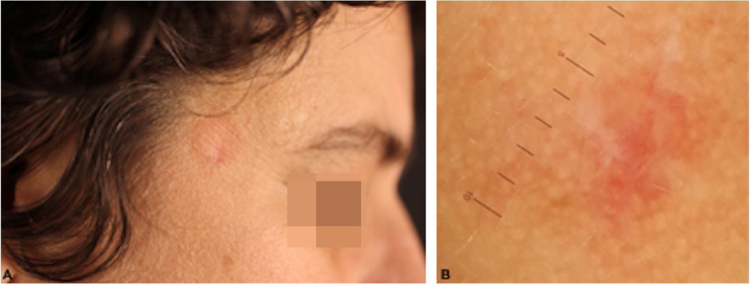
(A), Clinical aspect of the residual lesion after three weeks of clobetasol. (B), Aspect at contact dermoscopy without immersion after three weeks.

T-cell pseudolymphomas have an indolent course and may resolve spontaneously after biopsy. In the present case, the authors opted for the use of high-potency topical corticosteroids on the lesion, with a favorable evolution after three weeks (Fig. 4).

## Financial support

None declared.

## Authors’ contributions

Rodrigo Gomes Alves: Drafting and editing of the manuscript; critical review of the literature; critical review of the manuscript.

Patricia Mayumi Ogawa: Approval of the final version of the manuscript; design and planning of the study; drafting and editing of the manuscript; critical review of the literature; critical review of the manuscript.

Mílvia Maria Simões e Silva Enokihara: Collection, analysis, and interpretation of data; intellectual participation in propaedeutic and/or therapeutic conduct of studied cases.

Sergio Henrique Hirata: Approval of the final version of the manuscript; design and planning of the study; collection, analysis, and interpretation of data; effective participation in research orientation; intellectual participation in propaedeutic and/or therapeutic conduct of studied cases.

## Conflicts of interest

None declared.
